# Screening marine algae metabolites as high-affinity inhibitors of SARS-CoV-2 main protease (3CLpro): an in silico analysis to identify novel drug candidates to combat COVID-19 pandemic

**DOI:** 10.1186/s13765-020-00564-4

**Published:** 2020-11-21

**Authors:** Ghazala Muteeb, Adil Alshoaibi, Mohammad Aatif, Md. Tabish Rehman, M. Zuhaib Qayyum

**Affiliations:** 1 Department of Nursing, College of Applied Medical Science, King Faisal University, P.O. Box 400, Al-Ahsa, 31982 Saudi Arabia; 2 Department of Physics, College of Science, King Faisal University, P.O. Box 400, Al-Ahsa, 31982 Saudi Arabia; 3 Department of Public Health, College of Applied Medical Science, King Faisal University, P.O. Box 400, Al-Ahsa, 31982 Saudi Arabia; 4 Department of Pharmacognosy, College of Pharmacy, King Saud University, Riyadh, 11451 Saudi Arabia; 5 Department of Biochemistry and Molecular Biology, Eberly College of Science, The Pennsylvania State University, State College, USA

**Keywords:** SARS-CoV-2, Marine-derived compounds, Callophysin A, Molecular docking and simulation, Seaweeds

## Abstract

The recent dissemination of SARS-CoV-2 from Wuhan city to all over the world has created a pandemic. COVID-19 has cost many human lives and created an enormous economic burden. Although many drugs/vaccines are in different stages of clinical trials, still none is clinically available. We have screened a marine seaweed database (1110 compounds) against 3CLpro of SARS-CoV-2 using computational approaches. High throughput virtual screening was performed on compounds, and 86 of them with docking score <  − 5.000 kcal mol^−1^ were subjected to standard-precision docking. Based on binding energies (< − 6.000 kcal mol^−1^), 9 compounds were further shortlisted and subjected to extra-precision docking. Free energy calculation by Prime-MM/GBSA suggested RC002, GA004, and GA006 as the most potent inhibitors of 3CLpro. An analysis of ADMET (Absorption, Distribution, Metabolism, Excretion, and Toxicity) properties of RC002, GA004, and GA006 indicated that only RC002 (callophysin A, from red alga *Callophycus oppositifolius*) passed Lipinski’s, Veber’s, PAINS and Brenk’s filters and displayed drug-like and lead-like properties. Analysis of 3CLpro-callophysin A complex revealed the involvement of salt bridge, hydrogen bonds, and hydrophobic interactions. callophysin A interacted with the catalytic residues (His41 and Cys145) of 3CLpro; hence it may act as a mechanism-based competitive inhibitor. Docking energy and docking affinity of callophysin A towards 3CLpro was − 8.776 kcal mol^−1^ and 2.73 × 10^6^ M^−1^, respectively. Molecular dynamics simulation confirmed the stability of the 3CLpro-callophysin A complex. The findings of this study may serve as the basis for further validation by in vitro and in vivo studies.

## Introduction

SARS-CoV-2 (severe acute respiratory syndrome-coronavirus-2) is a member of the *Coronaviridae* family of the order Nidovirales and belongs to the genera of β-CoVs (beta-coronaviruses) [3, 33]. It is the 7th member of the HCoV (human coronavirus) family, which causes severe pneumonia-like infection in humans. The other six members of HCoVs which are responsible for respiratory and gastro-intestinal infection in humans are 229E, OC43, NL63, HKU1, SARS-CoV (severe acute respiratory syndrome-CoV), and MERS-CoV (middle east respiratory syndrome-CoV). While the four HCoVs, namely 229E, OC43, NL63, and HKU1, are not pathogenic and cause only mild infection, the other three, i.e., SARS-CoV, SARS-CoV-2, and MERS-CoV, are highly contagious and causes serve infection. The first case of SARS-CoV-2 has been reported in Dec 2019 in Wuhan city (Hubei province of China), and since then, it has spread across the globe [11, 31]. WHO (world health organization) has declared the spread of SARS-CoV-2 as a pandemic in Mar 2020 [4]. As of 31 Aug 2020, a total of 25,085,685 confirmed cases have been reported worldwide, with mortality of 843,927 (https://covid19.who.int).

The genome of SARS-CoV-2 contains a 29.9 kb long (+)-ssRNA containing 11 ORFs (open reading frames). At 5′ end of RNA, the ORFs 1a and 1b encode for polypeptides pp1a and pp1ab, respectively [25]. These polypeptides are cleaved into 16 nsps (non-structural proteins) like PLpro (nsp 3), 3CLpro or Mpro (nsp5), ssRNA binding protein (nsp9), growth factor-like protein (nsp10), RNA-dependent RNA polymerase (nsp12), RNA helicase (nsp13), exo-ribonuclease (nsp14), endo-ribonuclease (nsp15) and O-ribose methyltransferase (nsp16) [3]. Conversely, the 3` end of the viral genome encodes 12-nested ORFs of viral structural proteins such as S (spike protein), E (envelope protein), N (nucleocapsid protein), and other accessory proteins [19]. Amongst different viral proteins, the main protease, i.e., Mpro or 3CLpro, is indispensable for the survival of SARS-CoV-2 as it is directly or indirectly engaged in the replication and expression of viral genes [27]. Thus, 3CLpro or Mpro appears to be a good target for the design and development of drugs/inhibitors against SARS-CoV-2 [37].

Here, we screened a library of natural compounds derived from marine seaweeds to identify a potential inhibitor of 3CLpro employing computational approaches. Marine metabolites have diverse chemical space and display diverse biological activities such as anti-inflammatory, anti-infection, anti-cancer, anti-viral, and anti-microbial [9]. We applied three molecular docking protocols, namely high through virtual screening, standard precision (SP), and extra precision (XP) molecular dockings with increasing stringent parameters. Finally, the most potent inhibitor was identified based on free energy calculations. We found callophysin A as a potential inhibitor of SARS-CoV-2 main protease, i.e., 3CLpro. The stability and dynamic behavior of 3CLpro-callophysin A was also evaluated by molecular dynamics (MD) simulation. The physicochemical and ADMET (Absorption, Distribution, Metabolism, Excretion, and Toxicity) properties of callophysin A was determined using the SwissADME tool. The findings of this study may help the scientific community and pharmaceutical industries to use callophysin A as a scaffold and develop it into a highly potent inhibitor/drug against SARS-CoV-2.

## Materials and methods

### Preparation of ligands

A natural compounds library derived from marine seaweeds containing 1110 unique ligands was downloaded from Seaweed Metabolite Database [6]. Ligands were prepared by removing any salt molecules and generating multiple ionization states at pH 7.4 ± 2.0 using “Epik module in the LigPrep tool (Schrodinger-2018-4, LLC, NY, USA)” as reported earlier [20, 21]. For any ligand, a maximum of 32 stereoisomers was generated, and their energies were minimized by employing an OPLS3e force field.

### Preparation of 3CLpro target protein

The three-dimensional coordinates of SARS-CoV-2 3CLpro (PDB Id: 6LU7; resolution 2.16 Å) was downloaded from the PDB-RCSB databank [15]. The target protein was pre-processed before the virtual screening and molecular docking by adding missing H-atoms, defining bond orders, deleting any heterogeneous ligand and water molecules, employing “Protein preparation wizard (Schrodinger-2018-4, LLC, NY, USA)” as described previously [12, 20]. Any missing loops or side chains were modeled using “Prime (Schrodinger-2018-4, LLC, NY, USA)”. Finally, a network of hydrogen-bonds was generated, and the energy of the whole system was minimized using the OPLS3e force field. A grid box (88 × 88 × 88 Å) around the substrate-binding sites of 3CLpro was with the help of the “Receptor-grid generation tool (Schrodinger-2018-4, LLC, NY, USA)”.

### HTVS (high throughput virtual screening) and SP (standard-precision), and XP (extra-precision) molecular docking

Marine seaweed library was screened against 3CLpro target protein using high throughput virtual screening (HTVS) in “Glide (Schrodinger-2018-4, LLC, NY, USA)” as reported previously [8, 13]. The top-scoring 86 ligands (docking score < − 5.000 kcal mol^−1^) were subjected to SP docking, and then 9 of them with a docking score of less than -6.000 kcal mol^−1^ were subjected to XP docking using “Glide (Schrodinger-2018-4, LLC, NY, USA)”. Results were analyzed in “Maestro (Schrodinger-2018-4, LLC, NY, USA)”. Docking free energy (Δ*G*) was used to enumerate the binding affinity (*K*_d_) of the ligand towards 3CLpro using the following relation [28, 29].

$$ \Delta G = - RT{\text{ln}}K_{{\text{d}}} $$

where *R* was Boltzmann gas constant (= 1.987 cal/mol/K), and *T* was the temperature (= 298 K), respectively.

### Free energy calculation using Prime/MM-GBSA

The free energy of all the 9 ligands was determined with the help of the “Prime/MM-GBSA method (Schrodinger-2018-4, LLC, NY, USA)” as published earlier [10]. Briefly, the binding energy of 3CLpro-ligand complex was measured in generalized Born and implicit solvent model along with rotamer search algorithms and molecular mechanics force field, using the following relation. In free energy calculation, the atoms of ligands were set free while the atoms of 3CLpro were set rigid, and the 3CLpro-ligand poses were ranked according to free energies.

$$ \Delta G = E\left( {{\text{complex}}} \right)_{{{\text{minimized}}}} {-}\left[ {E\left( {{\text{ligand}}} \right)_{{{\text{minimized}}}} + \, E\left( {{\text{protein}}} \right)_{{{\text{minimized}}}} } \right] $$

### Physicochemical, drug-like, and ADMET properties

SwissADME was employed to determine the physicochemical, drug-like, and ADMET (Absorption, Distribution, Metabolism, Excretion, and Toxicity) properties of the most potential ligands (RC002, GA004, and GA006) as reported earlier [5, 14, 34].

### MD (molecular dynamics) simulation

MD simulation was performed to evaluate the dynamics and stability of 3CLpro-ligand complexes using “Desmond (Schrodinger-2018-4, LLC, NY, USA). MD simulation was performed in an orthorhombic box wherein the 3CLpro-ligand complex was placed at the center, at least 10 Å away from the box boundaries. The simulation box was solvated with TIP3P explicit water model and neutralized by adding counter ions. The physiological conditions were imitated by adding 0.15 mM NaCl. OPLS3e force field was employed to minimize the energy of the whole system with 2000 iterations keeping a convergence criterion of 1 kcal/mol/Å. Finally, a 50 ns production simulation was performed using the NTP ensemble at room temperature (298 K) and atmospheric pressure (1 bar). Nose–Hoover Chain thermostat and Matrtyna-Tobias-Klein barostat were employed to preserve constant temperature and pressure, respectively [2, 23]. The timestep was fixed at 2 fs, and energies and structures were recorded at every 10 ps in the trajectory.

## Results and discussion

### HTVS, SP, and XP molecular docking of marine seaweed compounds against 3CLpro

In structure-based drug design, HTVS and molecular docking are practical approaches to identify potential inhibitors of a protein from an extensive database of compounds [7, 18]. Here, we have utilized HTVS to screen a library of marine seaweed derived compounds against 3CLpro. In HTVS, around 50.5% compounds (i.e., 561) have been identified to bind to 3CLpro at the active site with varying binding energies (− 8.537 to − 0.254 kcal mol^−1^). We selected the compounds displaying docking score lower than − 5.000 kcal mol^−1^ (86 compounds) in HTVS and subjected them to SP docking (Additional file 1: Table S1). The compounds showing less than − 6.000 kcal mol^−1^ docking score in SP docking were identified as BZ004, GA004, GA005, GA006, GA007, RC002, RL497, RP011, and RR019; the details of their structure, source, and chemical nature is presented in Table 1. These 9 compounds were further screened by XP docking to identify the most potent inhibitor of 3CLpro. The XP docking scores of the shortlisted compounds were between − 4.897 and − 8.832 kcal mol^−1^ (Table 2). Other parameters such as Glide g-score, Glide e-model, and Glide energy of potential inhibitors of 3CLpro were in the range of − 4.970 to − 8.832 kcal mol^−1^, − 37.964 to − 92.771 kcal mol^−1^, and − 27.338 to − 69.594 kcal mol^−1^, respectively (Table 2). Further, the free energy of 3CLpro-inhibitor interaction was calculated using Prime/MM-GBSA on the shortlisted potential compounds. The free (Prime/MM-GBSA) energies of seaweed compounds were varied between − 37.64 to − 54.38 kcal mol^−1^ (Table 2). Based on the XP docking score and lowest free energy, the most potent inhibitors of 3CLpro were identified as RC002 (callophysin A), followed by GA004 (nigricanoside A), and GA004 (nigricanoside A dimethyl ester). callophysin A is abundantly isolated from *Callophycus oppositifolius* from Truant Island, Australia [26]. callophysin A has been reported to possess various biological activities such as anti-tumor agents [32] and insecticidal toxins [22]. Nigricanoside A and nigricanoside A dimethyl ester are isolated from green alga *Avraincillea nigricans*, abundantly found in Dominica, and have been reported to possess anti-cancer activities [36].

**Table 1:** Chemical identity and the source of marine seaweed compounds showing promising results against 3CLpro of SARS-CoV-2

S. no	Compound ID	Seaweed name	Structure of compound
1	BZ004	*Zonariu toumefortii*	5-[(1S,3Z)-1-hydroxyhex-3-en-1-yl]-8-[(2Z, 5Z)-10-oxo-10-(2,4,6-trihydroxyphenyl)deca-2,5-dien-1-yl]-2-phenyl-1H, 2H,3H,5H,8H-[1,2,4]triazolo[1,2-a]pyridazine-1,3-dione
2	GA004 (Nigricanoside A)	*Avrainvillea nigricans*	2,3-Dihydroxypropyl (5ξ)-6-O-[(4Z,8E, 13Z)-1-carboxy-10-{[(4Z, 9E)-15-carboxy-8,11-dihydroxy-4,9-pentadeca dien-7-yl]oxy}-11-hydroxy-4,8,13-nonadecatrien-7-yl]-α-L-arabino -hexopyranoside
3	GA005 (Nigricanoside B)	*Avrainvillea nigricans*	2,3-Dihydroxypropyl (5ξ)-6-O-[(4Z,8E,13Z, 16Z)-1-carboxy -10-{[(4Z,9E)-15-carboxy-8,11-dihydroxy-4,9-penta decadien-7-yl] oxy}-11-hydroxy-4,8,13,16-nonadecatetraen-7-yl]-α-L-arabino-hexopyranoside
4	GA006 (Nigricanoside A dimethyl ester)	*Avrainvillea nigricans*	2,3-Dihydroxypropyl 6-O-[(5Z,9E,14Z)-11-{[(4Z,9E)-8,11-di hydroxy-16-methoxy-16-oxo-4,9-hexadecadien-7-yl]oxy}-12-hydroxy-1-methoxy-1-oxo-5,9,14-icosatrien-8-yl]-β-D-galacto pyranoside
5	GA007 (Nigricanoside B dimethyl ester)	*Avrainvillea nigricans*	methyl (5Z,9E,14Z,17Z)-11-{[(4Z,9E)-8,11-dihydroxy-16-methoxy -16-oxohexadeca-4,9-dien-7-yl]oxy}-8-{[6-(2,3-dihydroxypropoxy)-3,4,5-trihydroxyoxan-2-yl]methoxy}-12-hydroxyicosa-5,9,14,17-tetraenoate
6	RC002 (Callophysin A)	*Callophycus oppositifolius*	2-[(4-hydroxyphenyl)methyl]-1H,2H, 3H,4H,9H-pyrido[3,4-b] indole-3-carboxylic acid
7	RL497	*Laurencia brongniartii*	4,6-dibromo-2,3-dimethanesulfinyl-1H-indole
8	RP011	*Polysiphonia lanosa*	3-bromo-5-(hydroxymethyl)benzene-1,2-diol
9	RR019	*Rhodomela confervoides*	methyl 4-{[({3-bromo-2-[(2,3-dibromo-4,5-dihydroxy phenyl) methyl]-4,5-dihydroxy phenyl}methyl)[(2,3-dibromo-4,5-dihydroxy phenyl)methyl]carbamoyl] amino}butanoate

**Table 2:** Molecular docking (extra-precision, XP) and free energy (Prime/MM-GBSA) parameters for the most promising inhibitors of SARS-CoV-2 3CLpro

S. no	Compound ID	XP docking score (kcal mol^−1^)	Glide g-score (kcal mol^−1^)	Glide e-model (kcal mol^−1^)	Glide energy (kcal mol^−1^)	Prime/MM-GBSA (kcal mol^−1^)
1	BZ004	− 5.804	− 6.037	− 75.022	− 61.010	− 39.25
2	GA004	− 8.073	− 8.073	− 92.771	− 68.680	− 51.01
3	GA005	− 6.560	− 6.560	− 78.373	− 61.810	− 42.35
4	GA006	− 8.832	− 8.832	− 90.809	− 68.794	− 49.67
5	GA007	− 7.858	− 7.858	− 83.276	− 69.594	− 38.65
6	RC002	− 8.776	− 8.234	− 63.993	− 39.208	− 54.38
7	RL497	− 6.236	− 6.240	− 51.698	− 37.200	− 50.12
8	RP011	− 4.897	− 4.970	− 37.964	− 27.338	− 47.25
9	RR019	− 6.142	− 6.536	− 80.199	− 61.819	− 37.64

Based on the XP docking score and free energy calculations by Prime-MM/GBSA, RC002 (Callophysin A), GA004 (Nigricanoside A), and GA006 (Nigricanoside A dimethyl ester) were selected for further analysis

### Investigation of physicochemical and ADMET properties

Determining physicochemical and ADMET properties employing computational approaches is a fast, robust, and accurate method [16]. We have evaluated the physicochemical and ADMET properties of RC002 (callophysin A), GA004 (nigricanoside A), GA006 (nigricanoside A dimethyl ester) using the SwissADME tool (Table 3). The physicochemical property, such as the molecular weight of callophysin A (322.36 g/mol), was within the tolerable 150–550 g/mol range of a drug-like molecule. Other physicochemical properties, such as the number of rotatable bonds, numbers of H-bond acceptors, and H-bond donors of callophysin A, were within the acceptable range. Conversely, the physicochemical properties of GA004 and GA006, such as molecular weight, number of rotatable bonds, numbers of H-bond donors, and H-bond acceptors, were beyond the acceptable limits. It is significant to notice that among RC002, GA004, and GA006, only RC002 possessed drug-like properties and obeyed different medicinal chemistry filters like Lipinski’s, Veber, PAINS, and Brenk filters. Moreover, RC002 demonstrated characteristic of a lead-like molecule with synthetic accessibility of 2.96 (Table 3). Thus, a detailed analysis of 3CLpro and callophysin A interaction along with molecular dynamics simulation was performed.

**Table 3:** Drug-like properties of RC002 (Callophysin A), GA004 (Nigricanoside A), and GA006 (Nigricanoside A dimethyl ester) using Swiss-ADME

Properties	Description	RC002 (Callophysin A)	GA004 (Nigricanoside A)	GA006 (Nigricanoside A dimethyl ester)
Physicochemical properties	Molecular weight	322.36 g/mol	873.08 g/mol	901.13
	Rotatable bonds	3	36	38
	H-bond acceptor	4	16	16
	H-bond donor	3	10	8
	Total polar surface area (TPSA)	76.56 Å^2^	273.36 Å^2^	251.36 Å^2^
Lipophilicity (log *P*_o/w_)	XlogP3	0.44	2.69	3.34
Solubility	log *S* (ESOL)	− 2.38 (Soluble)	− 4.57 (Moderately soluble	− 5.02 (Moderately soluble)
	log *S* (Ali)	− 1.62 (Very soluble)	− 8.08 (Poorly soluble)	− 8.30 (Poorly soluble)
Pharmacokinetics	GI absorption	High	Low	Low
	BBB permeability	Yes	No	No
	P-gp substrate	Yes	Yes	Yes
	CYP1A2 inhibitor	No	No	No
	CYP2C19 inhibitor	No	No	No
	CYP2C9 inhibitor	No	No	No
	CYP2D6 inhibitor	Yes	No	No
	CYP3A4 inhibitor	No	No	No
	Skin permeability (log *K*_*p*_)	− 7.95 cm/s	− 9.72 cm/s	− 9.43 cm/s
Drug-likeness	Lipinski	Yes; 0 violation	No; 3 violations	No; 3 violations
	Veber	Yes	No; 2 violations	No; 2 violations
	Bioavailability score	0.55	0.11	0.17
Medicinal chemistry	PAINS	1 alert: indol_3yl_alk	0 alert	0 alert
	Brenk	0 alert	1 alert; isolated alkene	2 alerts; isolated alkene; > 2 esters
	Lead-likeness	Yes	No; 2 violations	No; 2 violations
	Synthetic accessibility	2.96	9.45	9.71

### Interaction between 3CLpro and callophysin A

In recent times, 3CLpro has emerged as the most suitable target for drug designing and development. It plays an essentials role in the cleavage of polypeptides pp1a and pp1ab into separate functional protein molecules [1, 38]. The first X-ray crystal structure of 3CLpro (PDB Id: 6LU7) in complex with N3 polypeptide inhibitor has been recently reported [15]. The salient feature of the 3CLpro structure is that it comprises 306 amino acid residues arranged in three domains (I–III). Domain I encompasses amino acid residues 8–101, domain II spans amino acid residues 102–184, and domain III is spread in 201–303 amino acid residues. Domain II primarily contains an antiparallel beta-sheet structure, and domain III is arranged in five α-helices ordered predominantly into an antiparallel globular cluster. Domain III is linked to domain II through a loop formed by amino acid residues 185–200. Domains II and III harbor the substrate-binding site of 3CLpro in a deep pocket with a catalytic Cys41-His145 dyad, a characteristic feature observed in other coronaviruses also [27]. An analysis of 3CLpro-N3 X-ray crystal structure suggests that the Sγ-atom of Cys145 interacts with Cβ-atom of the vinyl group. The lactam at P1 of N3 inserts itself into the S1 subsite of 3CLpro, which is formed by the side chains of Phe140, Asn142, Glu166 and His163 (forms hydrogen bond with N3), and the main chains of Phe140 and Leu141 along with two water molecules [15]. Similarly, the Leu at the P2 site sits deeply into the hydrophobic S2 subsite, constituted by His41, Met49, Tyr54, Met165, and alkyl side of Asp187. The Val at the P3 site is exposed to the solvent, while Ala at the P4 site is enclosed in a small hydrophobic pocket created by the side chains of Met165, Leu167, Phe185, Gln192, and the main chain of Qln189. Further, the P5 site interacts with Pro168 and backbone of residues Ala191 and Gln192 through van der Waals interactions. The benzyl group interacts with Thr24 and Thr25 through van der Waals interactions in the S1′ site [15].

**Fig. 1:**
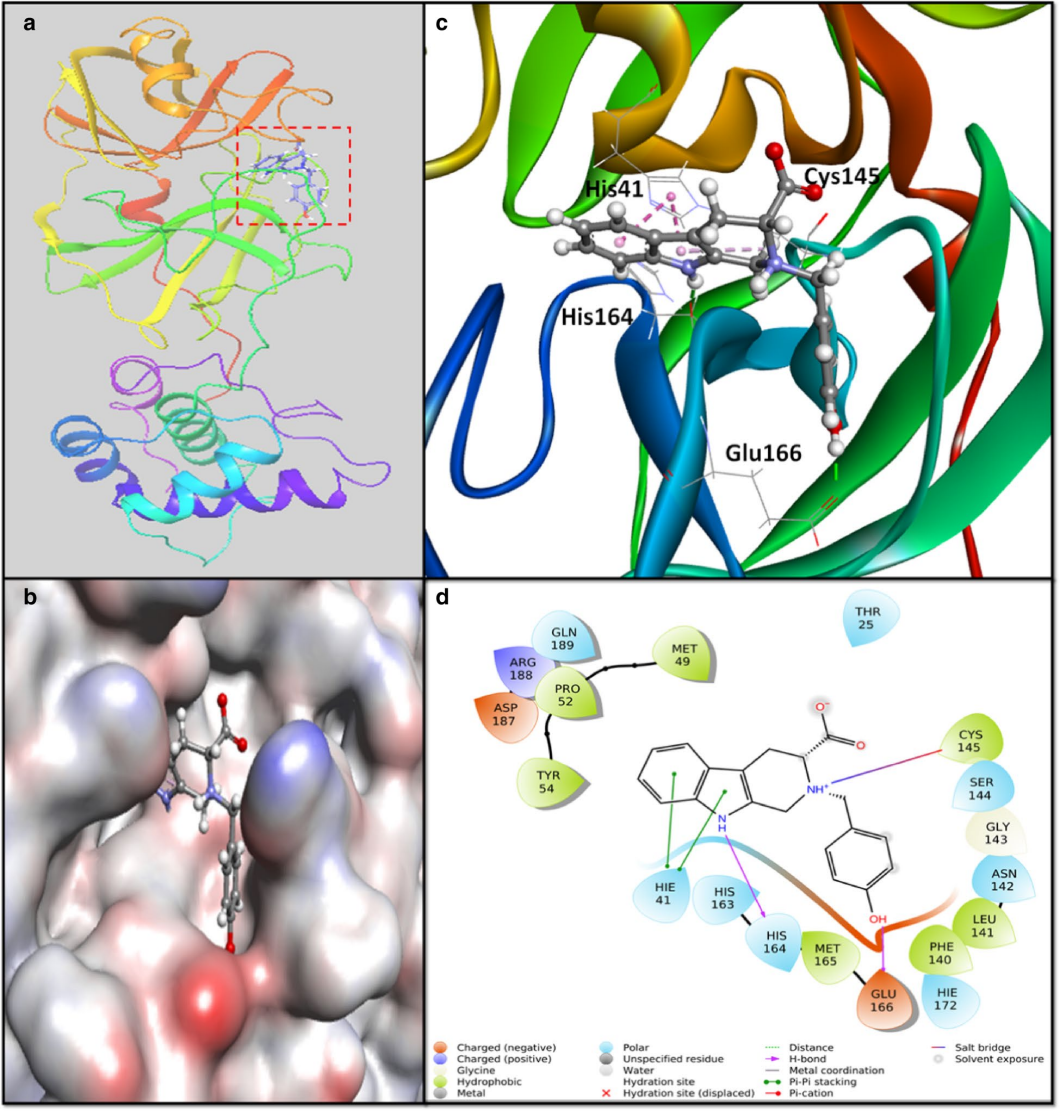
Molecular docking between callophysin A and 3CLpro in extra-precision (XP) mode. **a** 2D and **b** 3D representation of the binding mode of callophysin A to the active site of 3CLpro, **c** Interaction between callophysin A and active site residues, and **d** Interaction between 3CLpro and callophysin A, showing the involvement of different amino acid residues and the molecular forces between protein and inhibitor

An investigation of callophysin A-3CLpro molecular interaction revealed that callophysin A was bound to the active site of 3CLpro through multiple interactions such as salt bridges, hydrogen bonding, and hydrophobic interactions (Fig. 1). The active site residue His41 at S2 subsite formed two hydrophobic (Pi–Pi) interactions, while Cys145 formed a salt bridge with callophysin A. The residues at S1 subsite, such as Glu166 and His164, were engaged in hydrogen bonding with callophysin A. Further, amino acid residues like Thr25, Met49, Pro52, Tyr54, Phe140, Leu141, Asn142, Gly143, Ser144, His163, Met165, His172, Asp187, Arg188, and Gln189 stabilized the 3CLpro-callophysin A complex by forming van der Waals’ interaction. It is interesting to notice that callophysin A interacted with hydrophobic S2 subsite residues like Met49, Tyr54, Met165 and the alkyl side of Asp187, and S1′ site residue Thr25. Docking energy and the corresponding binding affinity of callophysin A towards 3CLpro were estimated as -8.776 kcal mol^−1^ and 2.73 × 10^6^ M^−1^, respectively (Table 1). Previous reports suggest the participation of active site residues His41 and Cys145 in the interaction with potential inhibitors such as ebselen, disulfiram, tideglusib, carmofur, shikonin, and PX-12 [15].

### MD (molecular dynamics) simulation analysis

MD is a powerful analysis tool to gain an insight into the structure and dynamics of proteins as a consequence of ligand/inhibitor binding. In this study, we have executed 50 ns MD simulation of 3CLpro-callophysin A complex, and the results are described as follows. The MD simulation of 3CLpro-GA004 and 3CLpro-GA006 complexes are presented in Additional file 1.

#### RMSD (root mean square deviation) calculations

**Fig. 2:**
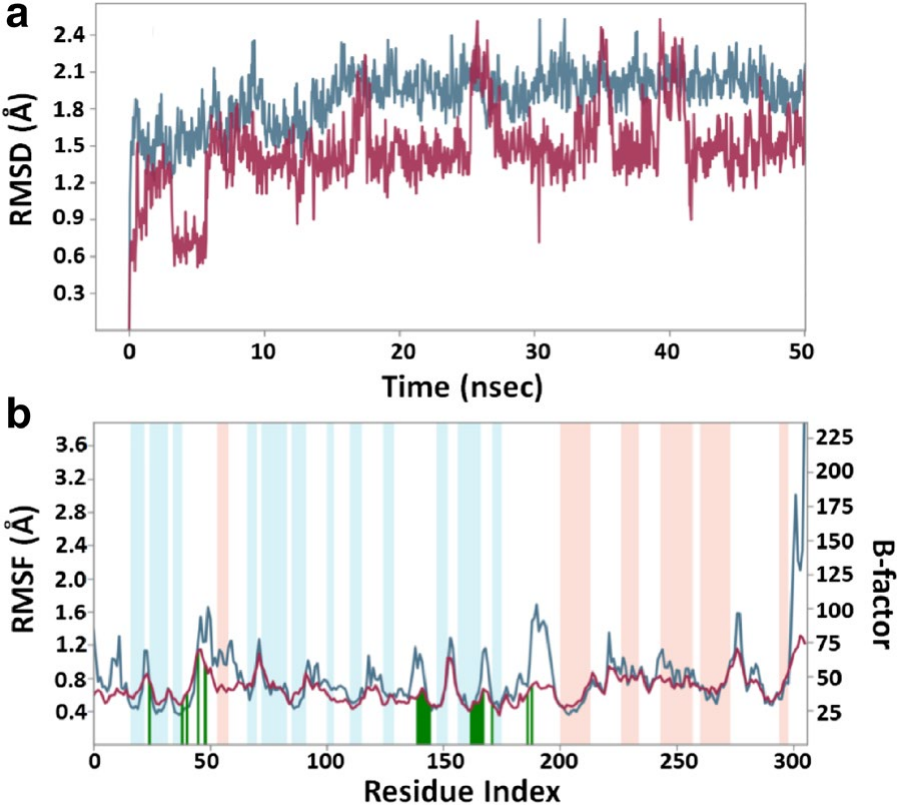
MD (Molecular dynamics) simulation of 3CLpro-callophysin A complex. **a** RMSD (Root mean square deviation) of 3CLpro alone (teal color) and in the presence of callophysin A (brown color), **b** RMSF (root mean square fluctuation) of 3CLpro in the presence of callophysin A (teal color), as compared with B-factor, which is determined during X-ray crystallography (brown color). Vertical green lines represent the location of amino acid residue forming an interaction with callophysin A. Light brown, and teal color bars represent the regions of α-helices and β-sheets

In MD simulation, RMSD is measured as a deviation in the structure of protein or protein–ligand complex as compared to a reference structure, usually the initial frame. Figure 2a shows RMSD in Cα-atoms of 3CLpro alone (teal color) or 3CLpro-callophysin A complex (brown line). As compared to the initial frame, no significant fluctuations were observed in RMSD values of protein as well as protein-inhibitor complex throughout the simulation time. The mean RMSD values of 3CLpro alone or in complex with callophysin A were obtained as 1.8979 and 1.5083 Å, respectively. Since the variation is RMSD values of protein and protein-inhibitor complex were much lower than the acceptable limit of 2.0 Å, the formation of a stable 3CLpro-callophysin A complex is anticipated (Fig. 2a).

#### RMSF (root mean square fluctuation) calculations

In MD simulation, the RMSF value of a protein is generally measured to access the fluctuations in side chains of the protein due to the binding of a ligand. Figure 2b depicted the RMSF of 3CLpro (teal color) in the presence of callophysin A during MD simulation and compared to the experimentally determined B-factor (brown color) obtained during X-ray crystallography. The vertical bars in brown, teal and white colors signify the α-helices, β-sheets, and loops regions of the protein, respectively. Moreover, green lines perpendicular to the X-axis demonstrate the identity of amino acid residues making an interaction with the inhibitor. There was a large fluctuation in the side chains of 3CLpro near the N-terminal end due to its unrestricted movement. Throughout the MD simulation, the RMSF values coincided with the B-factor values, except in loop regions. Minor fluctuations in RMSF values of 3CLpro side chains might be due to the entry and binding of callophysin A into the active site.

#### Protein–ligand interaction analysis

**Fig. 3:**
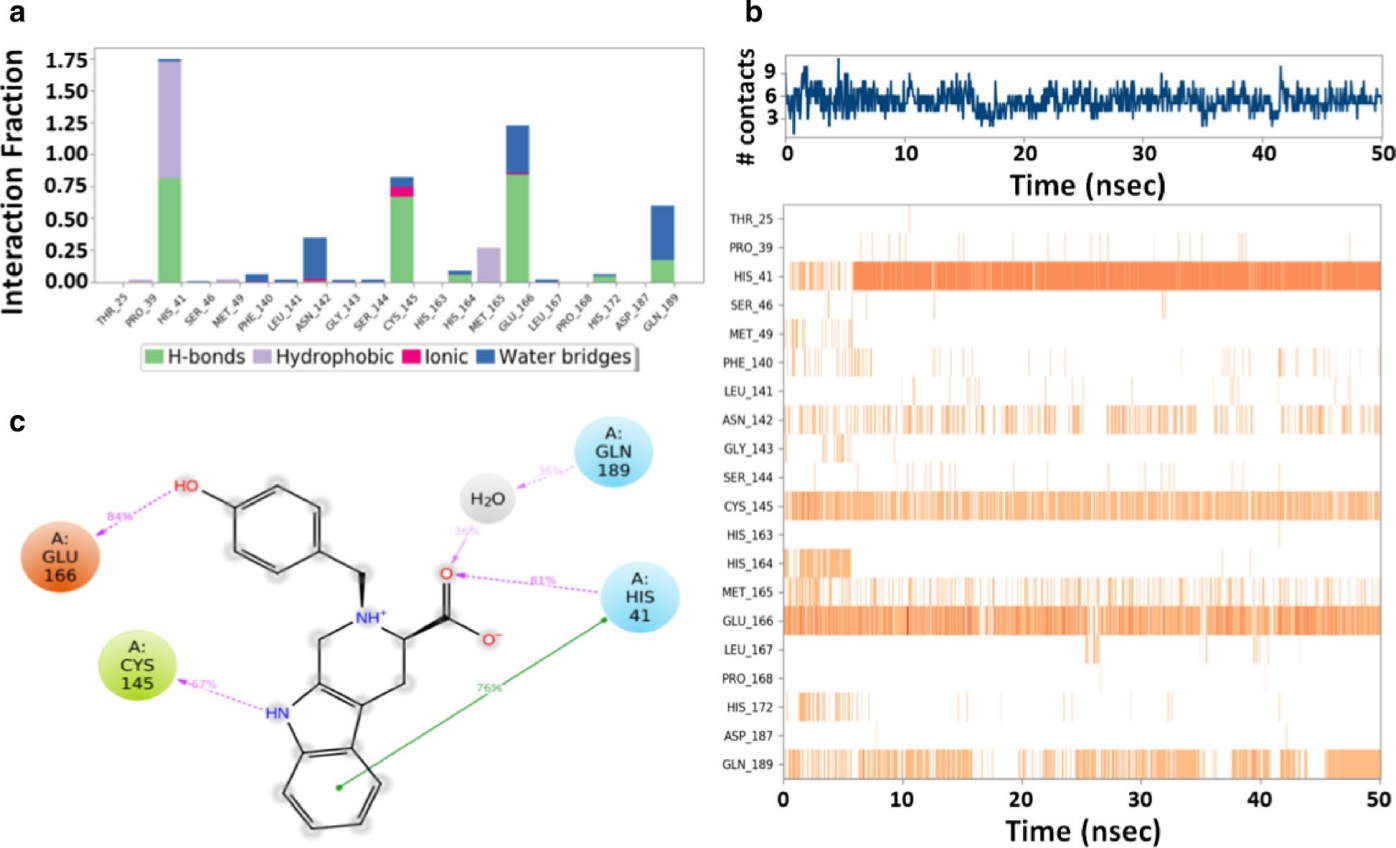
Interaction between 3CLpro and callophysin A during the simulation. **a** Amino acid residues of 3CLpro making different kinds of interaction with callophysin A, **b** *Upper panel:* variation in the number of contacts between 3CLpro and callophysin A during the simulation. *Lower panel:* participation of different amino acid residues in making contacts with callophysin A as a function of simulation, and **c** Percentage of simulation time for which some significant amino acid residues participate in the interaction with callophysin A

An investigation of 3CLpro-callophysin A interaction during MD simulation suggests that primarily hydrogen bonding was the driving force to stabilize the protein-inhibitor complex (Fig. 3a). The total number of contacts between 3CLpro and callophysin A during the simulation was determined to vary in the 2–10 range, with an average of 7 contacts (Fig. 3b, upper panel). Moreover, the involvement of amino acid residues in making contact with callophysin A during simulation showed that His41, Cys145, Glu166, and Gln189 were involved in making contact for most of the simulation (Fig. 3b, lower panel). The catalytic residue His41 of 3CLpro formed a hydrogen bond and hydrophobic interaction with callophysin A for 81% and 76% of simulation time, respectively. Similarly, another catalytic residue, Cys145, interacted with callophysin A through a hydrogen bond for and 67% simulation time. Another essential residue, Glu166, interacted with the inhibitor by forming a hydrogen bond for 84% simulation, while Gln189 formed a hydrogen bond through a water molecule for 36% simulation time (Fig. 3c).

#### Secondary structure analysis

**Fig. 4:**
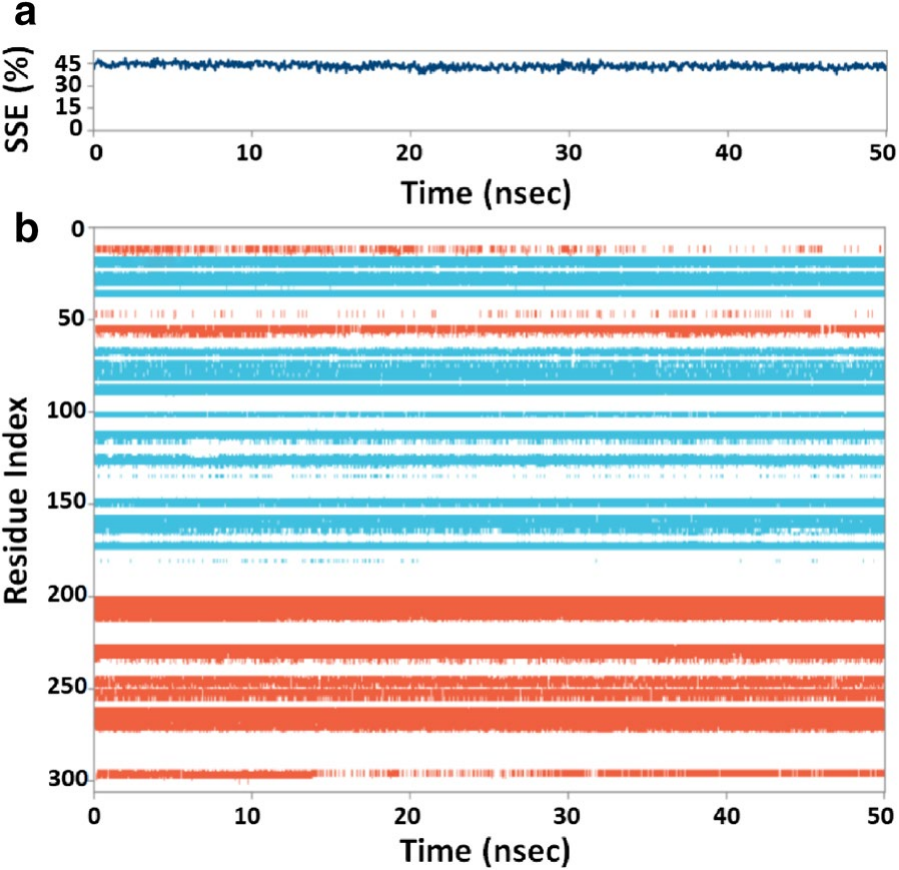
**a** Percentage of 3CLpro-callophysin A secondary structure element (SSE) varied during simulation, and **b** Involvement of 3CLpro amino acid residues in SSEs formation, wherein α-helices β-sheets are represented in light brown and teal colors, respectively

The interaction between a protein and an inhibitor may alter the secondary structure elements (SSE) of the protein. In this study, we monitored the changes in SSE of 3CLpro due to the binding of callophysin A during the simulation (Fig. 4). The total SSE of 3CLpro in complex with callophysin A was 43% (α-helix = 20% and β-sheets = 23%), which was in agreement with the reported values of SSEs 52% (27% α-helix and 25% β-sheets) (Fig. 4a, upper panel) [15]. The results indicate that the binding of callophysin A to 3CLpro did not considerably modify its secondary structure. Moreover, the participation of each amino acid residue in the SSE formation of 3CLpro as a function of simulation is also shown (Fig. 4a, lower panel).

#### Radius of gyration (rGyr) and different surface area analysis

**Fig. 5:**
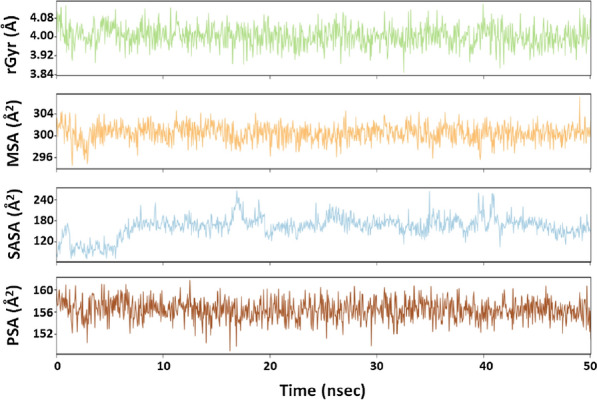
Variation in **a** rGyr (radius of gyration), **b** MSA (molecular surface area), **c** SASA (solvent accessible surface area), and **d** PSA (polar surface area) of 3CLpro-callophysin A complex during the simulation

The radius of gyration (rGyr) is considered a significant indicator of the protein’s folding state in different conditions. Here, the rGyr of 3CLpro-callophysin A complex was measured to gain an insight into the compactness of protein during the simulation (Fig. 5a). The rGyr of 3CLpro fluctuates between 3.85–4.14 Å, with an average value of 4.00 Å. The solvent-exposed surface area of a protein under different conditions is accessed to look for conformational changes [30]. Here, different surface area such as molecular surface area (MolSA), solvent accessible surface area (SASA), and polar surface area (PSA) of 3CLpro in complex with callophysin A were measured during the simulation to explore the exposure of the protein to solvent molecules and thus to access its conformational stability (Fig. 5b–d). MolSA, SASA, and PSA of callophysin A were in the range of 294.59–306.98 Å^2^, 81.32–155.52 Å^2^, and 152.28–159.46 Å^2^, respectively. The average values of MSA, SASA, and PSA were estimated to be 300.45 Å^2^, 162.53 Å^2^, and 156.37 Å^2^, respectively. Although the values of SASA fluctuated significantly for the initial part of the simulation (0–10 ns), it gets stabilized and remains within acceptable error once favorable contacts are made between protein and inhibitor. The results of rGyr and surface areas confirmed the formation of a stable 3CLpro-callophysin A complex.

## Conclusions

The utility of computational tools such as high throughput virtual screening, molecular docking, molecular dynamics simulation, and free energy calculation has been proven in the past for identifying inhibitors from a collection of ligand databases [17, 24, 35]. In the present study, a library of natural compounds derived from marine seaweed (1110 ligands) has been screened against 3CLpro employing different computational tools. After initial high throughput virtual screening, top-scoring ligands based on docking scores are further screened by standard-precision (SP) and extra precision (XP) molecular dockings against 3CLpro. Based on XP docking scores, 9 compounds, namely BZ004, GA004, GA005, GA006, GA007, RC002, RL497, RP011, and RR019, have been identified as the most promising inhibitors of the main protease 3CLpro. The binding poses of these 9 compounds have been further evaluated by free energy calculations using Prime-MM/GBSA. Based on XP docking energy and Prime/MM-GBSA energy, RC002, GA004, and GA006 have been identified as the most potent inhibitors of 3CLpro. Further, the analysis of physicochemical and ADMET properties of RC002, GA004, and GA006 lead to the identification of RC002 as the most promising molecule, which fulfills all the criteria of a drug-like, lead-like molecule with acceptable physicochemical and ADMET properties. RC002, also known as callophysin A, has been isolated from the red alga *Callophycus oppositifolius* in Australia. Chemically, RC002 or callophysin A is 2-[(4-hydroxyphenyl)methyl]-1H,2H, 3H,4H,9H-pyrido[3,4-b] indole-3-carboxylic acid. Analysis of 3CLpro-callophysin A interaction has shown that callophysin A interacts with the catalytic residues of 3CLro His41 and Cys145 through hydrophobic interactions and salt bridge, respectively. MD simulation of 3CLpro-callophysin A complex confirms the formation of a stable complex. The outcome of this study warrants further validation from in vitro and in vivo studies to confirm the effectiveness of callophysin A against 3CLpro.

## Supplementary information

**Additional file 1.** Additional tables and figures.

## Data Availability

All the data generated or analyzed during this study are included in this published article and its additional file.

## Abbreviations

SP docking: Standard-precision docking
XP docking: Extra-precision docking
RMSD: Root mean square deviation
RMSF: Root mean square fluctuation
MD: Molecular dynamics
ADMET: Absorption, Distribution, Metabolism, Excretion, and Toxicity
rGyr: Radius of gyration
SASA: Solvent accessible surface area
PSA: Polar surface area
MSA: Molecular surface area
MM-GBSA: Molecular mechanics-general Born surface area
OPLS: Optimized Potentials for Liquid Simulations

## Code availability

Different modules of Schrodinger-2018 (Schrodinger, LLC, NY, USA) have been employed.
