# Supplementary material for: Screening marine algae metabolites as high-affinity inhibitors of SARS-CoV-2 main protease (3CLpro): an in silico analysis to identify novel drug candidates to combat COVID-19 pandemic
Source: Appl Biol Chem. 2020 Nov 21;63(1):79. doi: 10.1186/s13765-020-00564-4 (PMC7680079; doi:10.1186/s13765-020-00564-4)
Supplement: Supplementary file 1 — Additional file 1. Additional tables and figures. [file 13765_2020_564_MOESM1_ESM.docx]

**Additional file 1: Data**

**Additional file 1: Table S1: Standard precision (SP) docking of best scoring compounds identified in high throughput virtual screening (HTVS)**

| **S. No.** | **Compound ID** | **Docking score**  **(kcal mol^-1^)** | **Glide g-score**  **(kcal mol^-1^)** | **Glide e-model**  **(kcal mol^-1^)** | **Glide energy**  **(kcal mol^-1^)** |
| --- | --- | --- | --- | --- | --- |
| 1. | GA004 | -8.537 | -8.537 | -101.516 | -72.335 |
| 2. | GA007 | -8.455 | -8.455 | -98.210 | -73.585 |
| 3. | GA005 | -8.291 | -8.291 | -75.055 | -59.515 |
| 4. | GA006 | -8.070 | -8.070 | -92.121 | -66.766 |
| 5. | RC002 | -7.699 | -8.157 | -60.740 | -39.422 |
| 6. | RR019 | -7.062 | -7.456 | -98.652 | -68.166 |
| 7. | RP011 | -6.682 | -6.755 | -33.321 | -21.888 |
| 8. | BZ004 | -6.658 | -6.890 | -75.266 | -56.545 |
| 9. | RL497 | -6.436 | -6.440 | -52.293 | -37.459 |
| 10. | RL114 | -5.971 | -5.971 | -40.421 | -30.832 |
| 11. | RG016 | -5.878 | -5.878 | -64.208 | -55.156 |
| 12. | RL460 | -5.832 | -5.832 | -25.477 | -22.830 |
| 13. | RL244 | -5.744 | -5.744 | -29.291 | -23.771 |
| 14. | GA011 | -5.735 | -7.471 | -85.571 | -62.203 |
| 15. | RL003 | -5.672 | -5.673 | -21.689 | -18.340 |
| 16. | RL502 | -5.671 | -5.671 | -20.552 | -17.727 |
| 17. | RJ006 | -5.653 | -5.653 | -43.506 | -33.937 |
| 18. | RL496 | -5.643 | -5.643 | -39.233 | -29.073 |
| 19. | RL147 | -5.614 | -5.614 | -45.813 | -35.667 |
| 20. | RL249 | -5.602 | -5.602 | -11.941 | -7.957 |
| 21. | RL476 | -5.598 | -5.598 | -42.914 | -35.600 |
| 22. | RR018 | -5.589 | -5.698 | -61.820 | -49.273 |
| 23. | RL462 | -5.560 | -5.560 | -41.434 | -32.972 |
| 24. | RL248 | -5.554 | -5.964 | -31.353 | -24.032 |
| 25. | RG009 | -5.551 | -5.551 | -38.461 | -30.173 |
| 26. | RP063 | -5.550 | -5.550 | -26.552 | -19.846 |
| 27. | RL499 | -5.545 | -5.546 | -64.972 | -49.381 |
| 28. | RL462 | -5.542 | -5.542 | -40.213 | -33.500 |
| 29. | RG012 | -5.524 | -5.524 | -43.446 | -33.330 |
| 30. | RL111 | -5.521 | -5.521 | -42.886 | -32.486 |
| 31. | RR068 | -5.510 | -5.571 | -34.482 | -26.578 |
| 32. | RL022 | -5.504 | -5.504 | -15.617 | -1.051 |
| 33. | RJ001 | -5.500 | -5.500 | -55.658 | -42.462 |
| 34. | RG008 | -5.490 | -5.490 | -40.832 | -33.801 |
| 35. | GA008 | -5.482 | -5.698 | -57.084 | -43.552 |
| 36. | RL194 | -5.469 | -5.469 | -45.400 | -37.615 |
| 37. | RL139 | -5.459 | -5.459 | -15.312 | -9.526 |
| 38. | RL529 | -5.440 | -5.440 | -27.887 | -22.399 |
| 39. | GA012 | -5.430 | -6.808 | -76.655 | -54.598 |
| 40. | BU002 | -5.425 | -5.425 | -22.184 | -17.255 |
| 41. | RG013 | -5.411 | -5.411 | -41.331 | -33.121 |
| 42. | RL443 | -5.406 | -5.406 | -38.211 | -30.977 |
| 43. | RL461 | -5.405 | -5.405 | -36.403 | -29.468 |
| 44. | RL146 | -5.396 | -5.396 | -40.715 | -32.525 |
| 45. | BT009 | -5.390 | -5.390 | -48.849 | -37.954 |
| 46. | RL007 | -5.387 | -5.387 | -8.810 | -9.619 |
| 47. | RL526 | -5.374 | -5.374 | -38.375 | -28.483 |
| 48. | RL029 | -5.344 | -5.344 | -36.062 | -29.761 |
| 49. | RC035 | -5.335 | -5.347 | -46.135 | -37.944 |
| 50. | RG006 | -5.315 | -5.315 | -37.835 | -30.785 |
| 51. | RL459 | -5.311 | -5.311 | -37.866 | -30.347 |
| 52. | RL151 | -5.310 | -5.310 | -33.445 | -26.252 |
| 53. | RP065 | -5.310 | -5.310 | -40.369 | -31.293 |
| 54. | RL171 | -5.304 | -5.325 | -46.848 | -35.492 |
| 55. | RL142 | -5.298 | -5.298 | -31.881 | -24.187 |
| 56. | GA009 | -5.294 | -5.526 | -57.302 | -42.524 |
| 57. | RG007 | -5.275 | -5.275 | -41.926 | -32.069 |
| 58. | RL423 | -5.269 | -5.269 | -44.521 | -33.776 |
| 59. | RL491 | -5.257 | -5.257 | -19.686 | -15.993 |
| 60. | RG010 | -5.229 | -5.229 | -44.829 | -36.922 |
| 61. | RJ007 | -5.221 | -5.221 | -29.004 | -34.250 |
| 62. | RJ004 | -5.211 | -5.211 | -42.395 | -41.788 |
| 63. | RL049 | -5.206 | -5.206 | -47.547 | -37.932 |
| 64. | RL437 | -5.200 | -5.200 | -18.600 | -14.614 |
| 65. | RP008 | -5.199 | -5.413 | -29.364 | -21.818 |
| 66. | RL193 | -5.195 | -5.195 | -46.382 | -38.567 |
| 67. | RL082 | -5.192 | -5.192 | -54.141 | -45.213 |
| 68. | RR069 | -5.189 | -5.278 | -37.443 | -28.721 |
| 69. | RR056 | -5.167 | -5.227 | -37.486 | -28.414 |
| 70. | RL464 | -5.165 | -5.165 | -38.525 | -29.953 |
| 71. | BT006 | -5.164 | -5.164 | -46.752 | -38.378 |
| 72. | RL495 | -5.161 | -5.162 | -50.690 | -38.419 |
| 73. | RL227 | -5.159 | -5.159 | -41.929 | -32.031 |
| 74. | BT007 | -5.151 | -5.151 | -19.909 | -19.934 |
| 75. | RL170 | -5.130 | -5.130 | -41.818 | -32.150 |
| 76. | RL030 | -5.089 | -5.089 | -34.493 | -28.257 |
| 77. | RR033 | -5.087 | -5.197 | -59.949 | -48.725 |
| 78. | RL444 | -5.071 | -5.073 | -10.551 | -13.356 |
| 79. | RL465 | -5.067 | -5.067 | -35.192 | -29.214 |
| 80. | RD026 | -5.062 | -5.062 | -38.177 | -29.883 |
| 81. | GC002 | -5.052 | -5.065 | -39.098 | -29.495 |
| 82. | RL463 | -5.047 | -5.047 | -31.480 | -25.073 |
| 83. | RR020 | -5.041 | -5.519 | -63.967 | -50.269 |
| 84. | RL050 | -5.008 | -5.008 | -41.794 | -35.071 |
| 85. | RL056 | -5.006 | -5.006 | -20.628 | -19.035 |
| 86. | RL247 | -5.001 | -5.001 | -13.230 | -12.393 |

**Computational studies of 3CLpro-GA004 and 3CLpro-GA006 interaction**

**Results and Discussion**

***Molecular docking analysis***

*Interaction between 3CLpro and GA004*

An insight into 3CLpro-GA004 interaction suggests that the ligand was bound to the substrate-binding site primarily through hydrogen bonding (**Additional file 1: Table S2**). The HD2 atom of active site residue His41 at S2 subsite formed a carbon-hydrogen bond with the O-atom of GA004. Another critical residue at the S1 subsite, namely Glu166:O, interacted with H19 and H30 of GA004 through two carbon-hydrogen bonds. Also, O-atoms of amino acid residues Leu141 and Thr190 formed carbon-hydrogen bonds with H26 and H44 of GA004, respectively (**Additional file 1: Figure S1**). Further, amino acid residues like Thr26, Thr45, Leu141, Gly143, Gln189, and Gln192 formed conventional hydrogen bonds with GA004. The amino acid residues like Thr24, Thr25, Leu27, Cys44, Ser46, Met49, Pro52, Tyr54, Phe140, Asn142, Ser144, Cys145, His163, His164, Met165, Leu167, Pro168, His172, Asp187, Arg188, and Ala191 stabilized the 3CLpro-GA004 complex by forming van der Waals’ interaction. Docking energy and the corresponding binding affinity of GA004 towards 3CLpro were estimated as -8.073 kcal mol^-1^ and 8.34 × 10^5^ M^-1,^ respectively (**Additional file 1: Table S2**).

*Interaction between 3CLpro and GA006*

An insight into 3CLpro-GA006 interaction suggests that the ligand was bound to the active site through hydrogen bonding and hydrophobic interactions (**Additional file 1: Table S2**). The active site residue His41 formed a Pi-Alkyl hydrophobic interaction with GA004. Another hydrophobic interaction (Alkyl) was formed between GA006 and Met165. The ligand GA006 interacted with 3CLpro through eleven conventional hydrogen bonding with Thr25:OG1, Cys44:O, Ser46:HN, Leu141:O, Asn142:HD21, Ser144:OG, Cys145:HN, Cys145:SG (two bonds), Gln189:HE21, and Thr190:HN (**Additional file 1: Figure S2**). The amino acid residues like Thr24, Thr26, Thr45, Met49, Phe140, Gly143, His163, His164, Glu166, Pro168, Asp187, Arg188, and Gln192 stabilized the 3CLpro-GA006 complex by forming van der Waals’ interaction. Docking energy and the corresponding binding affinity of GA006 towards 3CLpro were estimated as -8.832 kcal mol^-1^ and 3.00 × 10^6^ M^-1,^ respectively (**Additional file 1: Table S2**).

**Additional file 1: Table S1: Molecular docking parameters of 3CLpro-GA004 and 3CLpro-GA006 interactions**

| **Donor atom-Acceptor atom** | **Distance (Å)** | **Nature of interaction** | **XP docking energy, Δ*G* (kcal mol^-1^)** | **Binding affinity, *K*_d_ (M^-1^)** |
| --- | --- | --- | --- | --- |
| ***GA004*** | | | | |
| GLY143:HN - LIG:O  GLN189:HN - LIG:O  LIG:H - GLN192:O  LIG:H - LEU141:O  LIG:H - THR45:OG1  LIG:H - THR45:OG1  LIG:H - THR26:O  HIS41:HD2 - LIG:O  LIG:H19 - GLU166:O  LIG:H26 - THR190:O  LIG:H30 - GLU166:O  LIG:H44 - LEU141:O | 2.4695  2.8227  2.8961  2.0579  2.6835  1.7736  1.8937  2.5659  2.8913  2.5172  2.6535  3.0416 | Conventional Hydrogen Bond  Conventional Hydrogen Bond  Conventional Hydrogen Bond  Conventional Hydrogen Bond  Conventional Hydrogen Bond  Conventional Hydrogen Bond  Conventional Hydrogen Bond  Carbon Hydrogen Bond  Carbon Hydrogen Bond  Carbon Hydrogen Bond  Carbon Hydrogen Bond  Carbon Hydrogen Bond | -8.073 | 8.34 × 10^5^ |
| ***GA006*** | | | | |
| SER46:HN - LIG:O  ASN142:HD21 - LIG:O  CYS145:HN - LIG:O  CYS145:SG - LIG:O  CYS145:SG - LIG:O  GLN189:HE21 - LIG:O  THR190:HN - LIG:O  LIG:H - LEU141:O  LIG:H - SER144:OG  LIG:H - THR25:OG1  LIG:H - CYS44:O  LIG:C - GLU166:O  LIG:C - MET165  HIS41 - LIG:C | 2.5155  2.6054  2.5047  3.6119  3.2373  2.0727  2.6673  1.9932  2.7352  2.1799  2.8222  3.5281  4.4584  4.6717 | Conventional Hydrogen Bond  Conventional Hydrogen Bond  Conventional Hydrogen Bond  Conventional Hydrogen Bond  Conventional Hydrogen Bond  Conventional Hydrogen Bond  Conventional Hydrogen Bond  Conventional Hydrogen Bond  Conventional Hydrogen Bond  Conventional Hydrogen Bond  Conventional Hydrogen Bond  Carbon Hydrogen Bond  Hydrophobic (Alkyl)  Hydrophobic (Pi-Alkyl) | -8.832 | 3.00 × 10^6^ |

*
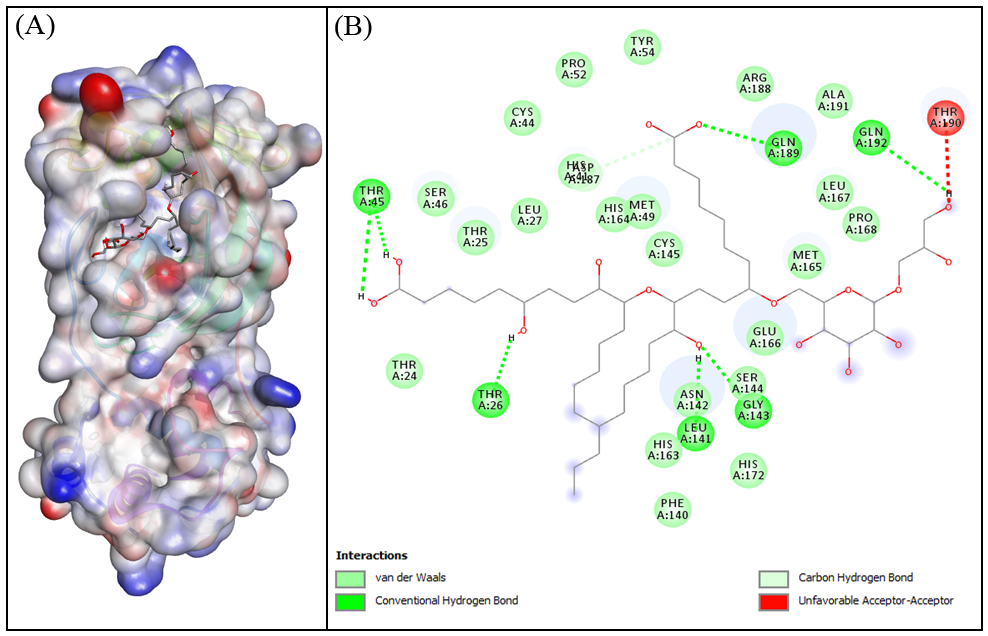
*

**Additional file 1: Figure SF1:** Molecular docking of GA004 at the substrate-binding site of 3CLpro. (A) Binding of GA004 at the active site cavity of 3Clpro, and (B) Molecular interaction between 3CLpro and GA004.

**
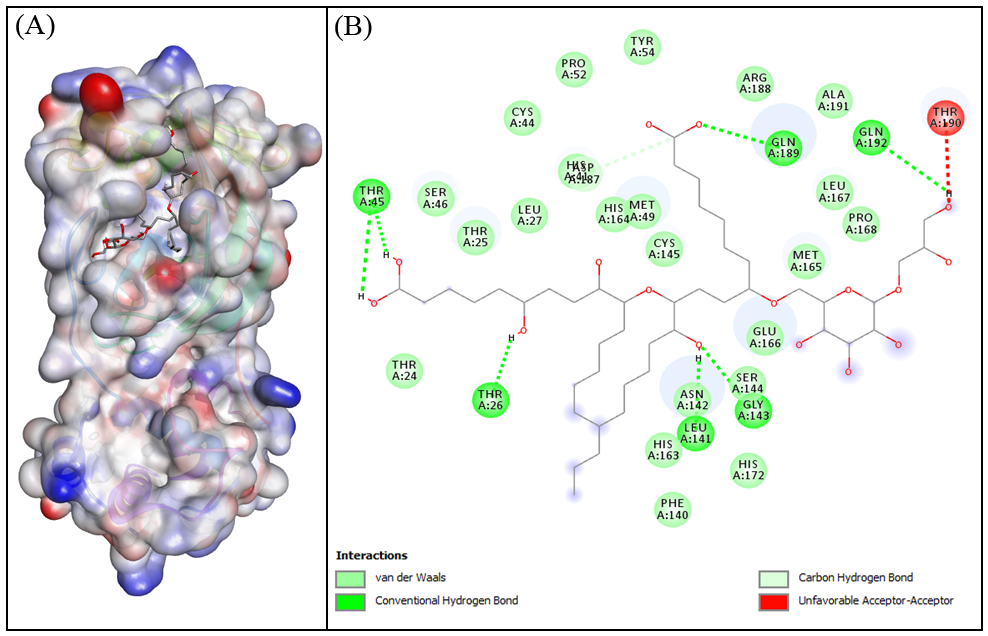
**

**Additional file 1: Figure SF2:** Molecular docking of GA006 at the substrate-binding site of 3CLpro. (A) Binding of GA006 at the active site cavity of 3Clpro, and (B) Molecular interaction between 3CLpro and GA006.

***Molecular dynamics simulation analysis***

*RMSD (root mean square deviation) calculations*

In MD simulation, RMSD is measured as a deviation in the protein or protein-ligand complex structure compared to a reference structure, usually the initial frame. **Additional file 1: Figure SF3A** shows RMSD in Cα-atoms of 3CLpro alone (black color) or in the presence of GA004 (red color) and GA006 (green color) as a function of simulation time. After initial fluctuations, the RMSD values of GA004 and GA006 stabilized in the last 20 ns simulation time. The mean RMSD values of 3CLpro alone or 3CLpro-GA004 and 3CLpro-GA006 complexes for the last 20 ns simulation were obtained as 2.1254, 1.4584, and 1.2032 Å, respectively. Since the variation is RMSD values of protein and protein-inhibitor complex were much lower than the acceptable limit of 2.0 Å, the formation of a stable 3CLpro-GA004 and 3Clpro-GA006 complexes is expected (**Additional file 1: Figure SF3A**).

*RMSF (root mean square fluctuation) calculations*

In MD simulation, the RMSF value of a protein is generally measured to access the fluctuations in the protein's side chains due to the binding of a ligand. Additional file 1: Figure S**F3B** depicts the RMSF of 3CLpro alone (black color) or in the presence of GA004 (red color) and GA006 (green color) during MD simulation. There was a large fluctuation in the side chains of 3CLpro near the N-terminal end due to its unrestricted movement. Throughout the MD simulation, RMSF values of GA004 and GA006 coincided with that of 3CLpro values, suggesting the formation of stable protein-ligand complexes. Minor fluctuations in RMSF values of 3CLpro side chains might be due to the entry and binding of prominent ligands such as GA004 and GA006 into the protein's active site.

*Analysis of radius of gyration (rGyr) and solvent accessible surface area (SASA)*

The gyration (rGyr) radius is considered a significant indicator of the protein’s folding state in different conditions. Here, the rGyr of 3CLpro in the absence (black color) and presence of GA004 (red color) and GA006 (green color) was measured to gain an insight into the compactness of protein during the simulation (**Additional file 1: Figure SF3C**). The average values of rGyr of 3CLpro alone, 3CLpro-GA004, and 3CLpro-GA006 were estimated to be 4.02, 4.18, and 4.11 Å, respectively. The solvent-exposed surface area of a protein under different conditions is accessed to look for any conformational changes. Here, solvent accessible surface area (SASA) of 3CLpro in the absence (black color) or as a complex with GA004 (red color) and GA006 (green color) was estimated to explore the exposure of the protein to solvent molecules and thus to access its conformational stability (**Additional file 1: Figure SF3D**). The average SASA values of 3CLpro alone, 3CLpro-GA004, and 3CLpro-GA006 complexes were 159.24, 168.83, and 163.57 Å^2^_,_ respectively. The results of rGyr and SASA confirmed the formation of stable 3CLpro-GA004 and 3CLpro-GA006 complexes.

**
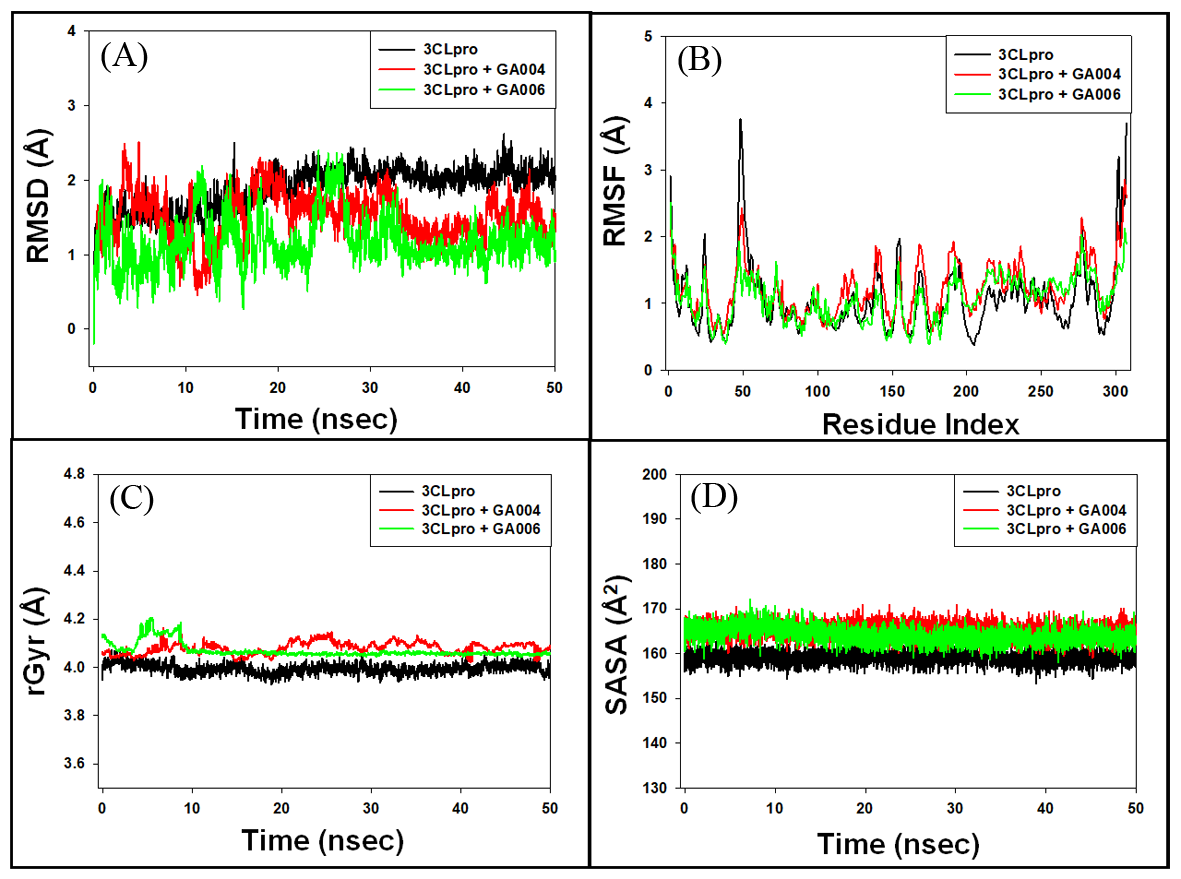
**

**Additional file 1: Figure SF3:** Molecular dynamics (MD) simulation of GA004 and GA006 with 3CLpro. (A) Dependence of root mean square deviation (RMSD) of 3CLpro alone, 3CLpro-GA004 and 3CLpro-GA006 complexes on the simulation time, (B) Root mean square fluctuation in Cα-atoms of 3CLpro alone or as a result of its interaction with GA004 and GA006, (C) Variation in the radius of gyration (rGyr) of 3CLpro-GA004 and 3CLpro-GA006 complexes as a function of simulation, and (D) Dependence of solvent accessible surface area (SASA) of 3CLpro alone, 3CLpro-GA004 and 3CLpro-GA006 complexes during the simulation.
